# Parvalbumin interneurons dysfunction is potentially associated with FαMNs decrease and NRG1-ErbB4 signaling inhibition in spinal cord in amyotrophic lateral sclerosis

**DOI:** 10.18632/aging.205351

**Published:** 2023-12-28

**Authors:** Qin Kang, Shishi Jiang, Jun Min, Fan Hu, Renshi Xu

**Affiliations:** 1 Department of Neurology, Medical College of Nanchang University, Nanchang 330006, Jiangxi, P.R. China; 2 Department of Neurology, Jiangxi Provincial People’s Hospital, First Affiliated Hospital of Nanchang Medical College, Clinical College of Nanchang Medical College, Nanchang 330006, Jiangxi, P.R. China; 3 Department of Neurology, The Second Affiliated Hospital of Nanchang University, Nanchang 330006, Jiangxi, P.R. China

**Keywords:** amyotrophic lateral sclerosis, parvalbumin, interneurons, FαMNs, ErbB

## Abstract

Objective: To investigate the alteration of PV interneurons in ALS mainly focusing its dynamic changes and its relationship with motor neurons and ErbB4 signaling.

Methods: SOD1G93A mice were used as ALS model. ALS animals were divided into different groups according to birth age: symptomatic prophase (50~60 days), symptomatic phase (90~100 days), and symptomatic progression (130~140 days). Immunofluorescence was performed for measurement of PV-positive interneurons, MMP-9, ChAT, NeuN and ErbB4. RT-qPCR and western blot were used to determine the expression of PV and MMP-9.

Results: PV expression was remarkably higher in the anterior horn of gray matter compared with posterior horn and area in the middle of gray matter in control mice. In ALS mice, PV, MMP-9 and ErbB4 levels were gradually decreased along with onset. PV, MMP-9 and ErbB4 levels in ALS mice were significantly down-regulated than control mice after onset, indicating the alteration of PV interneurons, FαMNs and ErbB4. SαMNs levels only decreased remarkably at symptomatic progression in ALS mice compared with control mice, while γMNs levels showed no significant change during whole period in all mice. MMP-9 and ErbB4 were positively correlated with PV. NRG1 treatment significantly enhanced the expression of ErBb4, PV and MMP-9 in ALS mice.

Conclusion: PV interneurons decrease is along with FαMNs and ErbB4 decrease in ALS mice.

## INTRODUCTION

As one of motoneuron (MN) spectrum of diseases, amyotrophic lateral sclerosis (ALS) is mainly characterized by the progressive death of MNs and early denervation of the neuromuscular junction [1–3]. As reported, the incidence of ALS is about 2/10,000 worldwide, with the mean onset age of 55~60 [4]. Patients with ALS usually suffer from muscle weakness, atrophy and paralysis, due to the retraction of MNs nerve terminals at striated muscles [5, 6]. Over 35% of ALS patients present with lesions on lower limb at the time of onset, while the upper limb and muscles for speaking and swallowing each occupy 30% of the cases, following with respiratory muscles <5% [7]. Patients with ALS usually appear respiratory failure in 2–5 years after onset, which may finally lead to the death [8].

Many factors may influence the incidence of ALS, including age, sex, hereditary factor, genetic mutations and etc. It is considered that males have higher incident rate of ALS than females [9]. Generally, SOD1 mutations are thought to be associated with the incidence of ALS, in which the neurons are hyperexcitable with an upregulated vulnerability to cell growth inhibition [10, 11]. Besides, many cellular dysfunctions are thought to be associated with ALS, such as oxidative and proteasome stress, excitatory toxicity due to abnormal glutamate signaling, mitochondrial dysfunction, cytoskeletal dysfunction and defects in axon transport, neuroinflammation, endoplasmic reticulum and protein folding stress [9, 12, 13]. However, the understanding for ALS, including both pathology and molecular mechanism, is far from enough. Currently, the treatment of ALS is still a huge clinical challenge [12, 14]. The application of neurotrophic drugs, the main treatment strategy, cannot provide an acceptable level of efficacy for most cases [15, 16].

The alteration of excitatory regulation of motoneurons in ALS is widely investigated. The change of exercise network excitability of motoneurons is considered as a main pathogenic mechanism of ALS [17, 18]. The changes of motoneurons, including αMNs and γMNs, play a key role in ALS development. The αMNs are further divided into two major categories as slow αMNs (SαMNs) and fast αMNs (FαMNs) along the anterior and posterior axes. Generally, FαMNs are considered as more sensitive to degrading in ALS [19]. It has been reported that the hyperexcitability of MNs can enhance their sensitivity to cell death through glutamatergic excitotoxicity, leading to the progressive dysfunction of MNs. Almost 1/3 of the spinal cord MNs may turned to hypoexcitatory in SOD1^G93A^ mice [20, 21]. The alteration of MNs from hyperexcitatory to hypoexcitatory reflects the importance of the balance of neurons’ excitability. In a recent study, it was found that in SOD1^G93A^ mice, MNs were reduced compared with the wild type animals [22].

Parvalbumin (PV) interneurons are a kind of calcium binding protein interneurons, as well as gamma amino butyric acid-ergic inhibitory interneurons [23]. PV interneurons can buffer calcium levels and protect neurons from excessive intracellular calcium [24]. The excitotoxicity induced by intracellular calcium overload is another key component of ALS progress, which is induced by hyperstimulation of neuronal glutamate receptors, resulting in activation of Ca^2+^-dependent enzyme pathway and finally leading to neuronal dysfunction and cell death [25]. In healthy individuals, PV interneurons regulate the balance of excitatory function of pyramidal neuronal populations in the brain and spinal cord through screening synaptic information and release of GABA [26]. Some early studies reported that the loss of motoneurons at early stage of ALS is mainly PV-negative interneurons, while the loss of PV-highly positive interneurons was markedly restricted [27, 28]. Studies have also observed the reduction of PV positive neurons and neuropeptide Y positive neurons in the motor cortex of deceased ALS patients, along with decrease of calcium binding protein positive neurons in cortex V and VI and a declining trend of calmodulin positive neurons [29, 30]. Besides, in other neurological diseases such as Alzheimer’s disease, PV interneurons were found to play a protect role in disease process [31]. However, up to now, few studies reported the details for the changes of PV interneurons in spinal cord during the ALS progression.

Erb-B2 receptor tyrosine kinase 4 (ErbB4) belongs to the family of ErbB transmembrane receptor tyrosine kinase, highly expressing in PV interneurons, especially in the cell bodies and proximal dendrites. ErbB4 is essential to maintain the normal function of PV interneurons, and the defection of ErbB4 may lead to reduced levels of activity dependent PV interneurons [32]. Besides, ErbB4 can be activated by NRG1, a nutritional factor containing epidermal growth factor like domains, in PV interneurons, maintaining neuronal excitation balance through regulation of γ-aminobutyric acid (GABA) [33, 34]. ErbB4 in PV-positive interneurons is also critical for NRG1 in long-term potentiation by enhancing GABA release [35]. In early studies, it has been already proven that ErbB4 can not only regulate the function of PV interneurons, but also influence the incidence of ALS [36]. In ALS, studies found ErbB4 was down-regulated along with reduced autophosphorylation [36, 37]. However, studies reporting the alterations of FαMNs and ErbB4 in ALS are still inadequate, and whether these alterations are related to the changes of PV interneurons is not known.

In the present study, we used an *in vivo* mouse ALS model to investigate the alteration of PV interneurons in ALS, mainly focusing its dynamic changes and its relationship with motor neurons and ErbB4 signaling. This study might provide deeper insights for PV interneurons dysfunction in ALS development, as well as provide novel research targets for ALS treatment.

## MATERIALS AND METHODS

### Animals and grouping

Non-transgenic B6SJL mice, and transgenic mice expressing human SOD1^G93A^ were obtained from the Nanjing Model Animal Research Center. We used the male SOD1^G93A^ mice (B6SJL-Tg SOD1^G93A^ 1Gur/NJU mice, 60 days) and female non-transgenic B6SJL mice to breed the next generation. The neonatal mice with SOD1^G93A^ mutation were used as the ALS model. The mice without SOD1^G93A^ mutation were used as the control. The polymerase chain reaction (PCR) reaction was used for the definition of SOD1^G93A^ mutation which was described below. The mice were maintained at Jiangxi Provincial People’s Hospital (No. 0001, 2021-12-12). All mice were housed in a condition of SPF and kept on a 12-hour light/dark cycle with access to food and water provided ad libitum. The protocols for animal care and use of laboratory animals were approved by Jiangxi Provincial People’s Hospital (No. 0001, 2021-12-12).

The ALS animals were divided into the different groups according to the birth age: the symptomatic prophase (50~60 days), symptomatic phase (90~100 days), and the symptomatic progression (130~140 days). The non-transgenic mice with the same age period were used as the control. Half male and half female mice were in all groups (*n* = 6 in each group). For the treatment of NRG1, 1.0 μg/ml recombinant NRG1 (Sciencell, China) dissolved in PBS was given by intranasal administration with different doses of 0.125, 0.25 and 0.5 mg/kg [38]. The mice received treatment of recombinant NRG1 every day from 50 days to 90 days. The control mice were given by the same volume of PBS.

The mice at the different age were euthanized by abdominal aorta blood collection after anesthesia under diethyl ether. The spinal cord was collected after normal saline cardiac perfusion and/or 4% paraformaldehyde perfusion. Besides, brain tissue was also collected in some experiments.

### Rotarod test

After 90 days, all animals underwent a weekly rotarod test to assess their motor function until 140 days. The test was conducted using an accelerating rotarod treadmill (Med Associates, USA), with the animals placed on the rotating rod, positioned 16.5 cm above a platform. In each test session, the mice were acclimated to the rotating rod, which spun at a constant speed of 4 revolutions per minute (rpm). Subsequently, the rod’s speed was gradually increased from 4 rpm to 40 rpm at a rate of 4 rpm per minute. The experiment lasted for 180 seconds, and the time it took for the animals to fall from the platform was recorded as the latency in the rotarod test or until the end of the experiment.

### Immunofluorescence

Immunofluorescence was performed for the measurement of PV-positive interneurons, matrix metalloproteinase-9 (MMP-9), choline acetyltransferase (ChAT), neuronal marker neuronal nuclei (NeuN) and ErbB4. Briefly, the spinal cord was collected, fixed with 4% paraformaldehyde, embedded with paraffin, sliced, dewaxed and hydrated with ethanol. The citric acid buffer was then added, following with PBS wash for three times. After blocked with 5% bovine serum albumin (BSA) for 30 minutes at 37°C, the samples were incubated with the primary antibodies of anti-PV (1/250, ab181086, Abcam, UK), anti-MMP-9 (1/5000, ab228402, Abcam), anti-ChAT (1/100, ab181023, Abcam), anti-NeuN (1/100, ab177487, Abcam) and anti-ErbB4 (1/500, ab19391, Abcam) overnight at 4°C. Then, samples were incubated with the corresponding secondary antibody (ab205719, Abcam) for 45 minutes at 37°C. DAPI was used for redyeing nucleus. A CKX53 laser scanning confocal microscope (Olympus, Japan) was used to take the photomicrographs. Briefly, every sixth section covering all the target cells was included (7~8 sections per animal), counted by a 400× lens. The cell ratio was determined using ImageJ software (Rasband, NIH, USA) through the calculation of cell areas. The atlas of spinal cord of SOD1^G93A^ C57BL/6J mice was used as a reference.

### Reverse transcription-quantitative PCR (RT-qPCR)

RT-qPCR was used to determine the mRNA expression of PV, MMP-9 and ErbB4. TRIzol reagent (Takara, Japan) was applied to extract total RNA from the spinal cord. The RNA concentration was determined using a NP80 spectrophotometer (NanoPhotometer). A HiScript II Q RT SuperMix (R223-01, Vazyme, China) was applied to synthesize the first-strand cDNA. The ChamQ Universal SYBR qPCR Master Mix (Vazyme) was used for PCR reaction in a Bio-Rad CFX96 fluorescent quantitative PCR instrument (Bio-Rad, China). RT-qPCR was repeated three times as follows: 95°C for 10 minutes, followed by 40 cycles (95°C for 10 seconds, 58°C for 30 seconds and 72°C for 30 seconds). The results were calculated performing 2–ΔΔCT (2-DeltaDelta CT) method. β-actin was applied for normalization. The primers (synthesized by Shanghai Sangon Biotech, China) were as follows: PV: forward, 5′-GGCCTCTGCTCATCCAAGTT-3′ and reverse, 5′-GAATGGACCCCAGCTCATCC-3′; MMP-9: forward, 5′-GGACCCGAAGCGGACATTG-3′ and reverse, 5′-CGTCGTCGAAATGGGCATCT-3′; ErbB4 forward, 5′-AGGAAAGATGGCAACTTTGGAC-3′ and reverse, 5′-ATCGGCCAGTGCAAGACTTAT-3′; β-actin: forward, 5′-AGGGAAATCGTGCGTGAC-3′ and reverse, 5′-CATACCCAAGAAGGAAGGCT-3′. β-actin was used as the internal control.

For the definition of SOD1^G93A^ gene in the transgenic mice, the DNA of mice tail was extracted using a mouse tail genomic DNA extraction kit (CoV in Biotechnology Co., Ltd., China) according to the manufacturer’s instruction. The primer for SOD1^G93A^ was provided by the Nanjing Model Animal Research Center with sequences of: upstream: forward, 5′-CTAGGCCACAGAATTGAAAGATCT-3′ reverse, 5′-CATCAGCCCTAATCCATCTGA-3′, downstream: forward, 5′-GTAGGTGGAAATTCTAGCATCATCC-3′, reverse, 5′-CGCGACTAACAATCAAAGTGA-3′. The amplification reaction was conducted using a 2× Taq PCR MasterMix (Solarbio, China) according to the manufacturer’s instruction in a TC-EA PCR amplification instrument (Bori Technology Co., Ltd., China). The PCR reaction was conducted under the condition of: 95°C for 5 minutes, 35 cycles of 94°C for 30 seconds, 60°C for 60 seconds and 72°C for 60 seconds, then 72°C for 120 seconds. The gel electrophoresis was scanned in an ultraviolet lamp box.

### Western blotting

RIPA buffer (Applygen Technology Co., Ltd., China) was applied to extract total protein from the spinal cord. The protein content was measured using a BCA protein kit (E-BC-K318-M, Elabscience, USA). Then, SDS-PAGE gel (5~15%) was applied to separate the proteins (8 μg per lane), and then proteins were transferred from gel to PVDF (IPVH00010, Millipore, USA) membranes. Primary antibodies of anti-PV (ZB-2301, 1/2000, ZSGB-Bio, China), anti-MMP-9 (TA336901, 1/2000, ZSGB-Bio), anti-ErbB4 (TA804610, 1/2000, ZSGB-Bio) or anti-β-actin (TA-09, 1/2000, ZSGB-Bio) were added to incubate the membranes at 4°C overnight after membranes were blocked by 3% non-fat milk (Applygen) containing 1× TBST for 1 hour. Then, horseradish peroxidase (HRP)-conjugated secondary goat anti-mouse/rabbit IgG antibody (1/500, Affinity, USA) was used to treat the membranes at 37°C for 45 minutes. An ultra-high sensitivity chemiluminescence imaging system (Bio-Rad) was applied to scan the membranes. The ImageJ software (Rasband, NIH, USA) was used to analyze the blots.

### Statistical analysis

All experiments were performed in triplicate. Differences were analyzed using Student’s *t*-test to analyze the comparison between two groups, and the comparisons between multiple groups were investigated by using one-way analysis of variance (ANOVA) followed by Tukey’s test (Graphpad Prism7). Pearson’s correlation was used for correlation analysis. A significant difference was indicated by the result of *P* < 0.05.

### Availability of data and materials

The data that support the findings of this study are available from the corresponding author.

## RESULTS

### PV interneurons was decreased in the spinal cord of ALS mice along with the onset time

**Figure 1:**
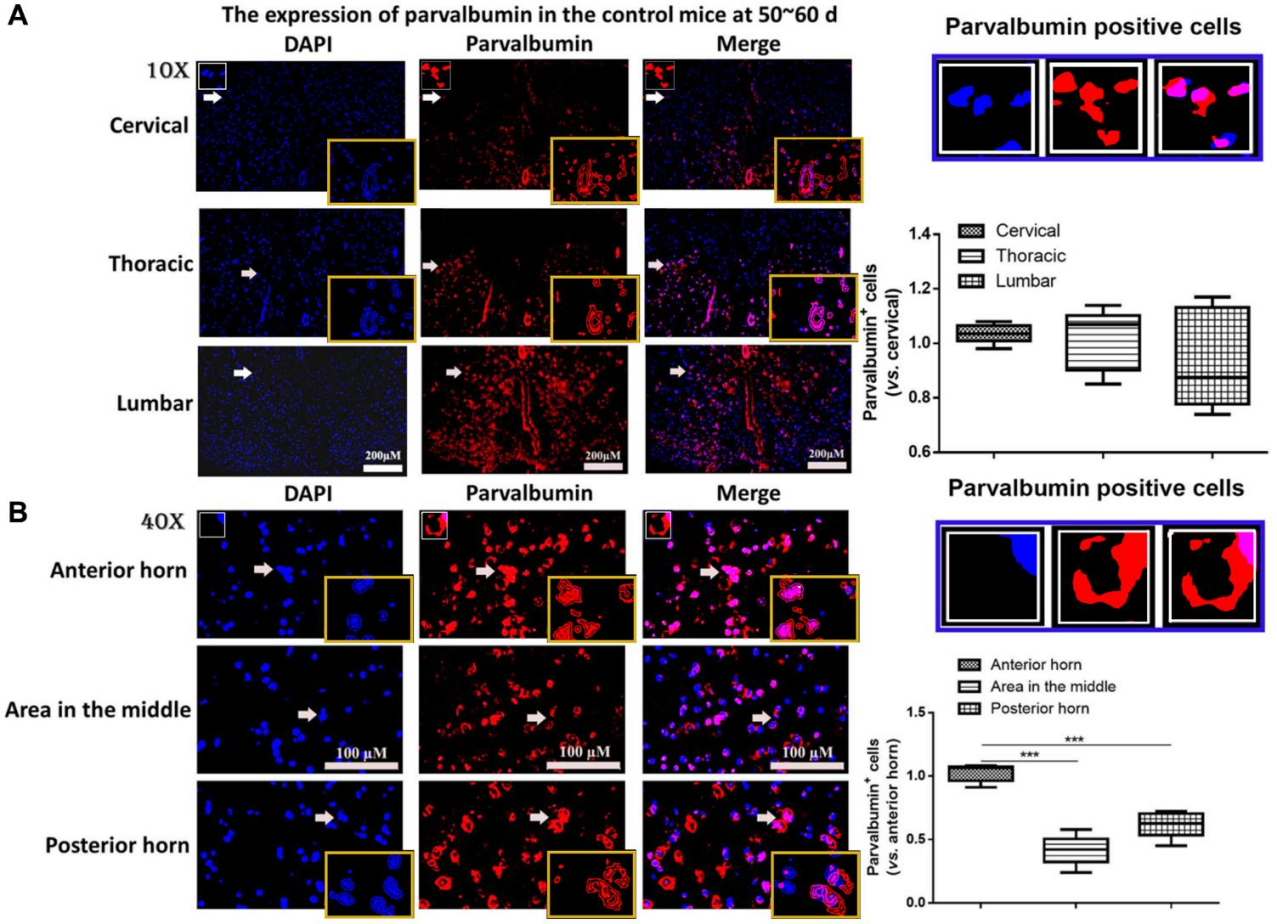
**The expression of parvalbumin (PV) in different positions of spinal cord.** (**A**) The expression of PV in cervical, thoracic and lumbar segments in the control mice. Scale bar = 200 μM. The yellow boxes represent the typical PV positive cells (enlarged approximate twice on the original position). The typical PV positive cells are enlarged approximate 4 times and shown on the top right panel. (**B**) The expression of PV in the anterior horn of gray matter, posterior horn and the area in the middle of gray matter in the control mice. The yellow boxes represent the typical PV positive cells (enlarged approximately twice on the original position). The typical PV positive cells are enlarged approximately 4 times and shown on the middle right panel. Scale bar = 100 μM. ^***^P < 0.001. In all experiments, half male and half female mice were used in each group (*n* = 6).

First the mutation of SOD1^G93A^ in B6SJL-Tg SOD1^G93A^ 1Gur/NJU mice was determined. In all 179 neonatal mice, 87 mice were with and 92 mice were without SOD1^G93A^ mutation. The mice were randomly divided into different groups with half male and half female mice (*n* = 6 in each group). To determine the role of PV interneurons in ALS, first, the expression of PV interneurons was measured in different of positions of spinal cord from control mice at 50~60 days. As shown in Figure 1A, the expression of PV in cervical, thoracic and lumbar segments showed no significant difference. The spinal cord in lumbar segments was then used. It was found the expression of PV was remarkably higher in the anterior horn of gray matter compared with the posterior horn and the area in the middle of gray matter in the control mice at 50~60 days (*P* < 0.05, Figure 1B). Thus, the anterior horn of gray matter in lumbar segments of the spinal cord was used for further studies.

**Figure 2:**
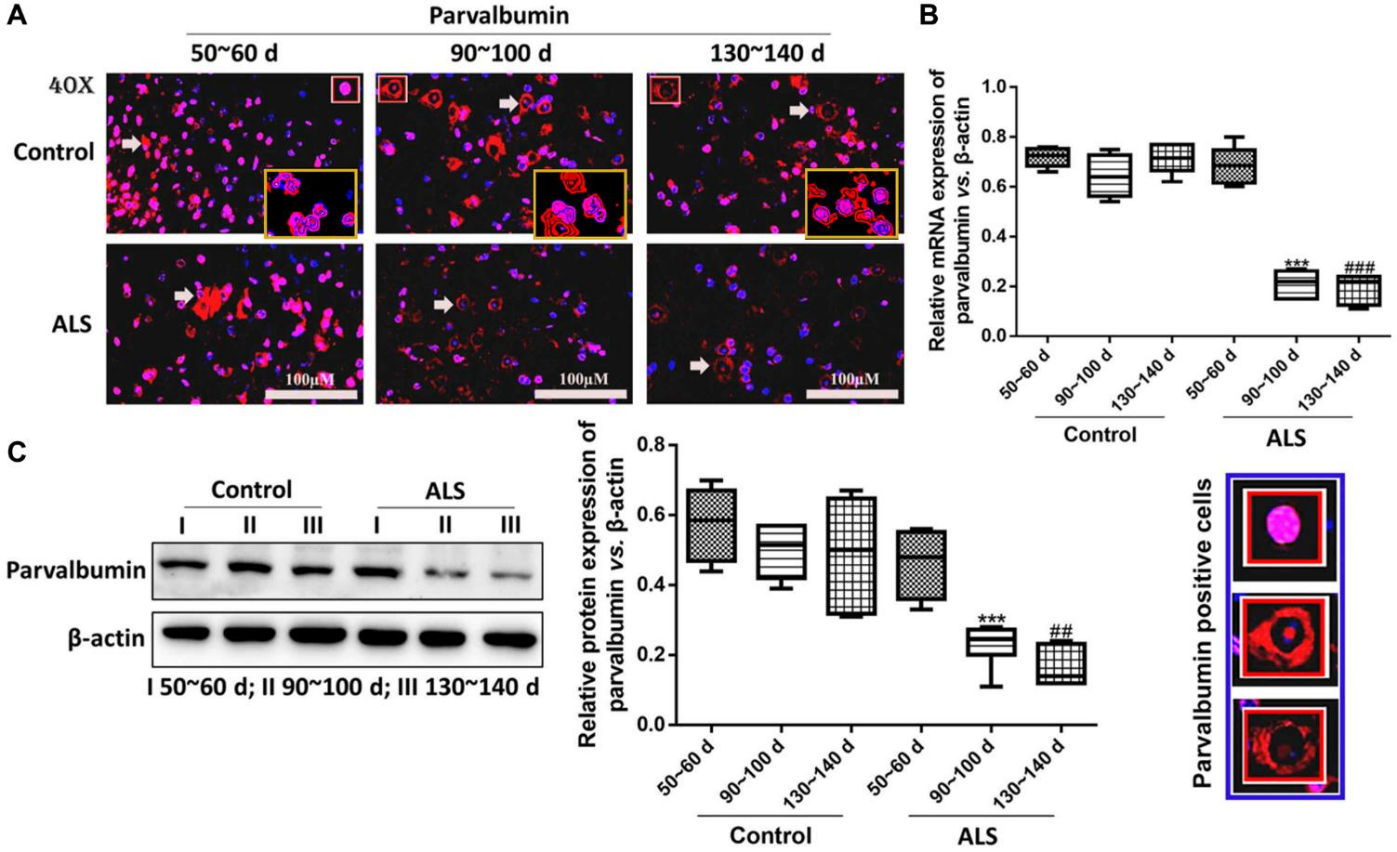
**PV interneurons in the spinal cord of ALS mice with different age by immunofluorescence.** (**A**) The yellow boxes represent the typical positive cells (enlarged approximate twice on the original position). The typical PV positive cells are enlarged approximate 4 times and shown on the bottom right panel. Scale bar = 100 μM. PV interneurons in the spinal cord of ALS mice with different age by RT-qPCR (**B**) and western blot (**C**). ^***^P < 0.001 vs. control at 90~100 days. ^###^*P* < 0.001 vs. control at 130~140 days. ^##^*P* < 0.01 vs. control at 130~140 days. In all experiments, half male and half female mice were used in each group (*n* = 6).

In ALS mice, the levels of PV interneurons were gradually decreased along with the onset time and the expression of PV was significantly declined at symptomatic phase (90~100 days) and the symptomatic progression (130~140 days) compared with the symptomatic prophase (50~60 days) (*P* < 0.05, Figure 2A). For the control mice, the change of PV was fluctuating, but the PV levels were all markedly higher in the control mice compared with the ALS mice after onset (all *P* < 0.05). The results of RT-qPCR and western blotting also provided similar results, in which the PV levels in the ALS mice were significantly down-regulated than the control mice after onset (all *P* < 0.05, Figure 2B, 2C). The mRNA and protein levels of PV also gradually decreased in ALS mice along with the onset time. Besides, we conducted a western blotting analysis and observed that the expression of PV in both frontal cortex and hippocampal tissue was also decreased in ALS mice along with the time, which was also significantly reduced at 90~100 d and 130~140 d compared with the control mice (Supplementary Figure 1).

### Different motoneurons in the spinal cord of ALS mice with different onset time

**Figure 3:**
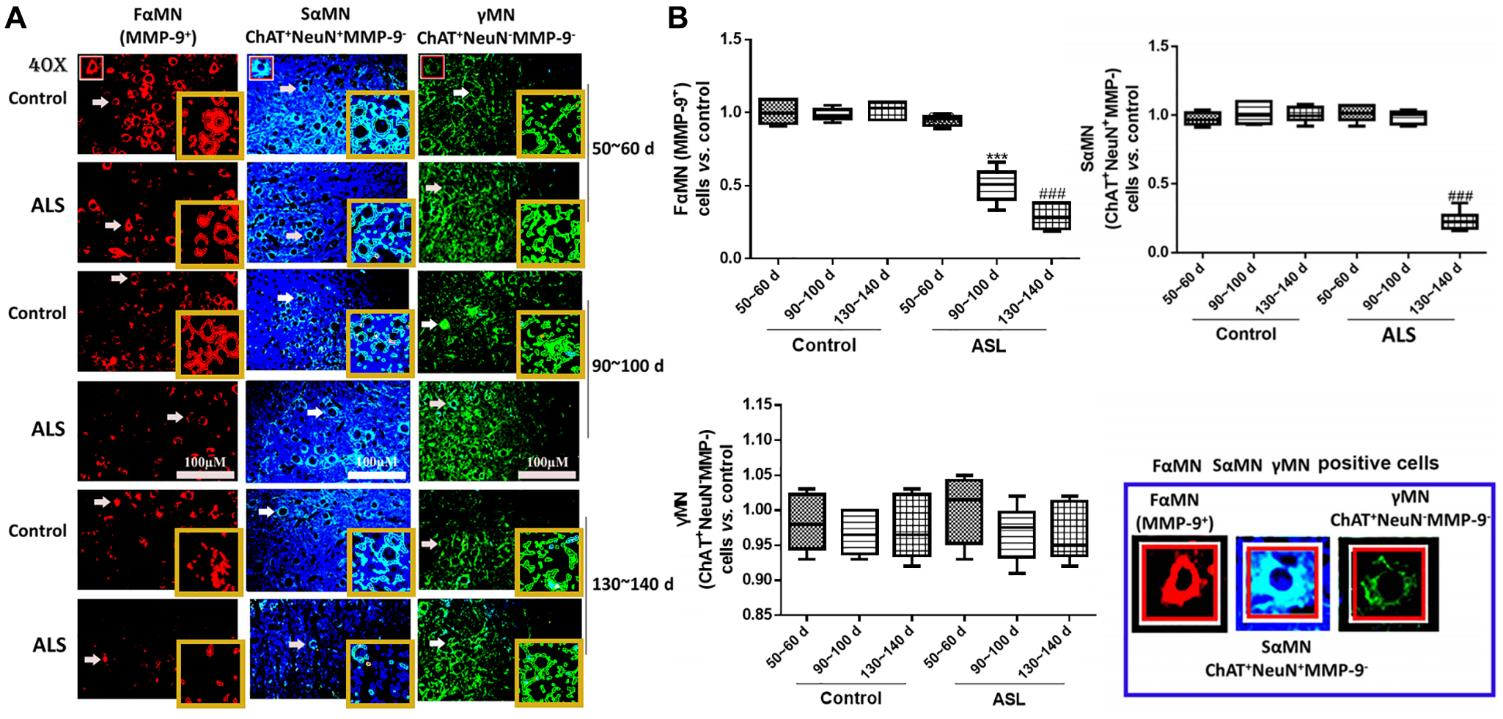
**Different motoneurons in the spinal cord of ALS mice with different onset time.** Different MNs are stained with different markers and colors, FαMNs (MMP-9^+^), SαMNs (ChAT^+^NeuN^+^MMP-9^−^) and (ChAT^+^NeuN^−^MMP-9^−^), with MMP-9^+^ (red), ChAT^+^ (green) and NeuN^+^ (blue). Scale bar = 100 μM (**A**) and the quantitative results (**B**). The yellow boxes represent the typical positive cells (enlarged approximate twice on the original position). The typical MMP-9^+^, ChAT^+^NeuN^+^MMP-9^−^ and ChAT^+^NeuN^−^MMP-9^−^ positive cells are enlarged approximately 4 times and shown on the bottom right panel. ^&&&^*P* < 0.001 vs. control at 50~60 days. ^***^P < 0.001 vs. control at 90~100 days. ^###^*P* < 0.001 vs. control at 130~140 days. In all experiments, half male and half female mice were used in each group (*n* = 6).

Then, the contents of different motoneurons in the spinal cord of ALS mice were detected. In this study, we found that the levels of FαMNs (MMP-9^+^) in ALS mice decreased significantly along with the onset time (Figure 3A, 3B). An additional result of FαMNs with marker of MMP-9^+^ and ChAT^+^ is also provided in Supplementary Figure 2. At symptomatic phase (90~100 days) and symptomatic progression (130~140 days), the levels of FαMNs were markedly declined in the ALS mice compared with the control mice (*P* < 0.05). For SαMNs (ChAT^+^NeuN^+^MMP-9^−^), the levels in ALS mice didn’t change significantly at symptomatic prophase (50~60 days) and symptomatic phase (90~100 days), while the levels of SαMNs decreased remarkably at symptomatic progression (130~140 days) in ALS mice compared with the control mice (*P* < 0.05). However, the levels of γMNs (ChAT^+^NeuN^-^MMP-9^−^) showed no significant change during the whole period in all mice.

### PV interneurons were positively correlated with MMP-9 in the spinal cord of ALS mice with different onset time

**Figure 4:**
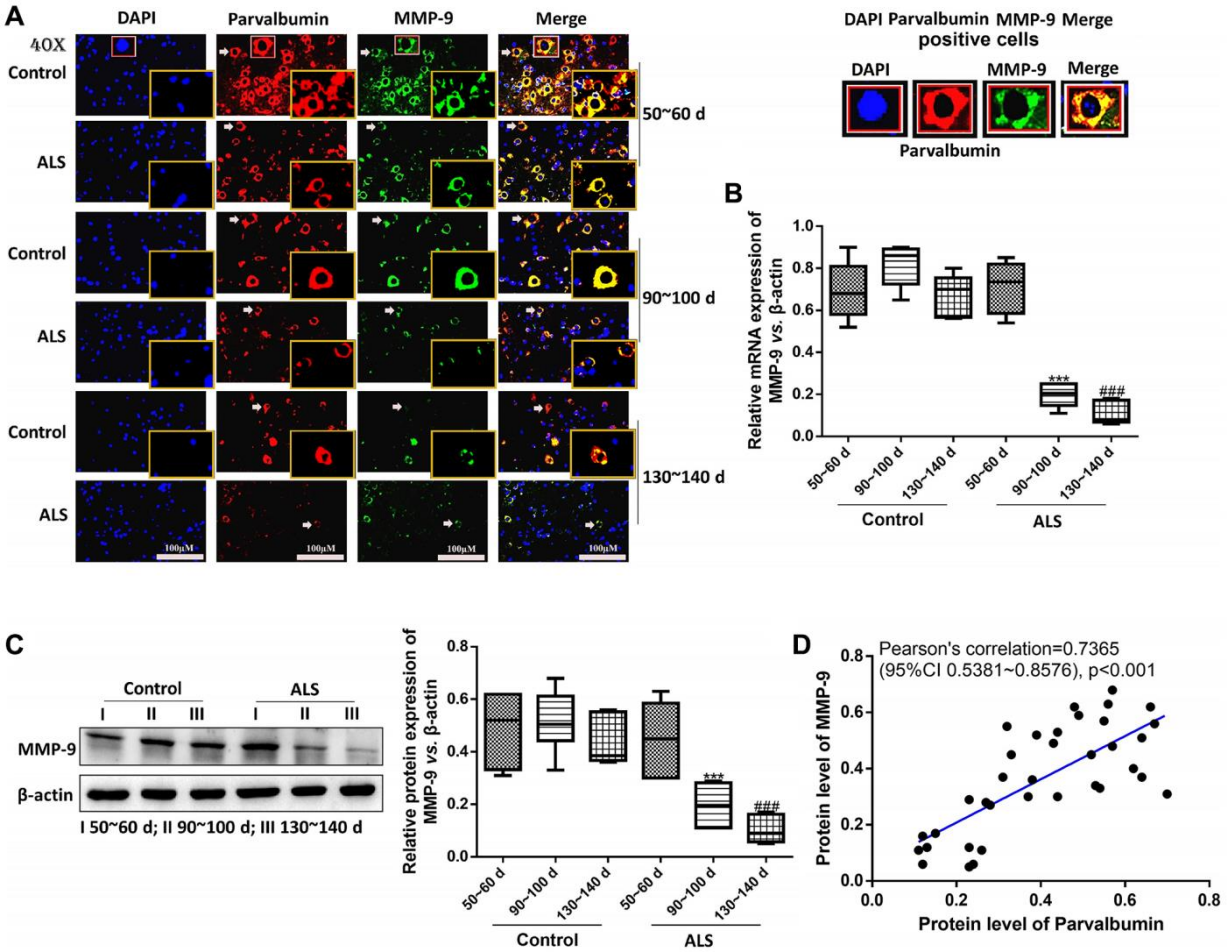
**PV interneurons were positively correlated with MMP-9 in the spinal cord of ALS mice with different onset time.** The expression of PV and MMP-9 tested by immunofluorescence. The yellow boxes represent the typical positive cells (enlarged approximately twice on the original position). The typical PV and MMP-9 positive cells are enlarged approximately 4 times and shown on the top right panel. Scale bar = 100 μM (**A**). The expression of MMP-9 was tested by RT-qPCR (**B**) and western blot (**C**). Pearson’s correlation for protein levels of PV and MMP-9 in all animals (control and ALS) (**D**). ^***^P < 0.001 vs. control at 90~100 days. ^###^*P* < 0.001 vs. control at 130~140 days. In all experiments, half male and half female mice were used in each group (*n* = 6).

Next, the mRNA and protein levels of MMP-9 were determined. It was found that both mRNA and protein levels of MMP-9 were decreased in ALS mice along with the onset time. Meanwhile, all levels of MMP-9 were significantly down-regulated in ALS mice compared with the control mice after onset (*P* < 0.05, Figure 4A–4C). Further Pearson’s correlation showed the protein levels of MMP-9 were positively correlated with the levels of PV (Figure 4D).

### The expression of ErbB4 was positively correlated with PV interneurons in the spinal cord of ALS mice

**Figure 5:**
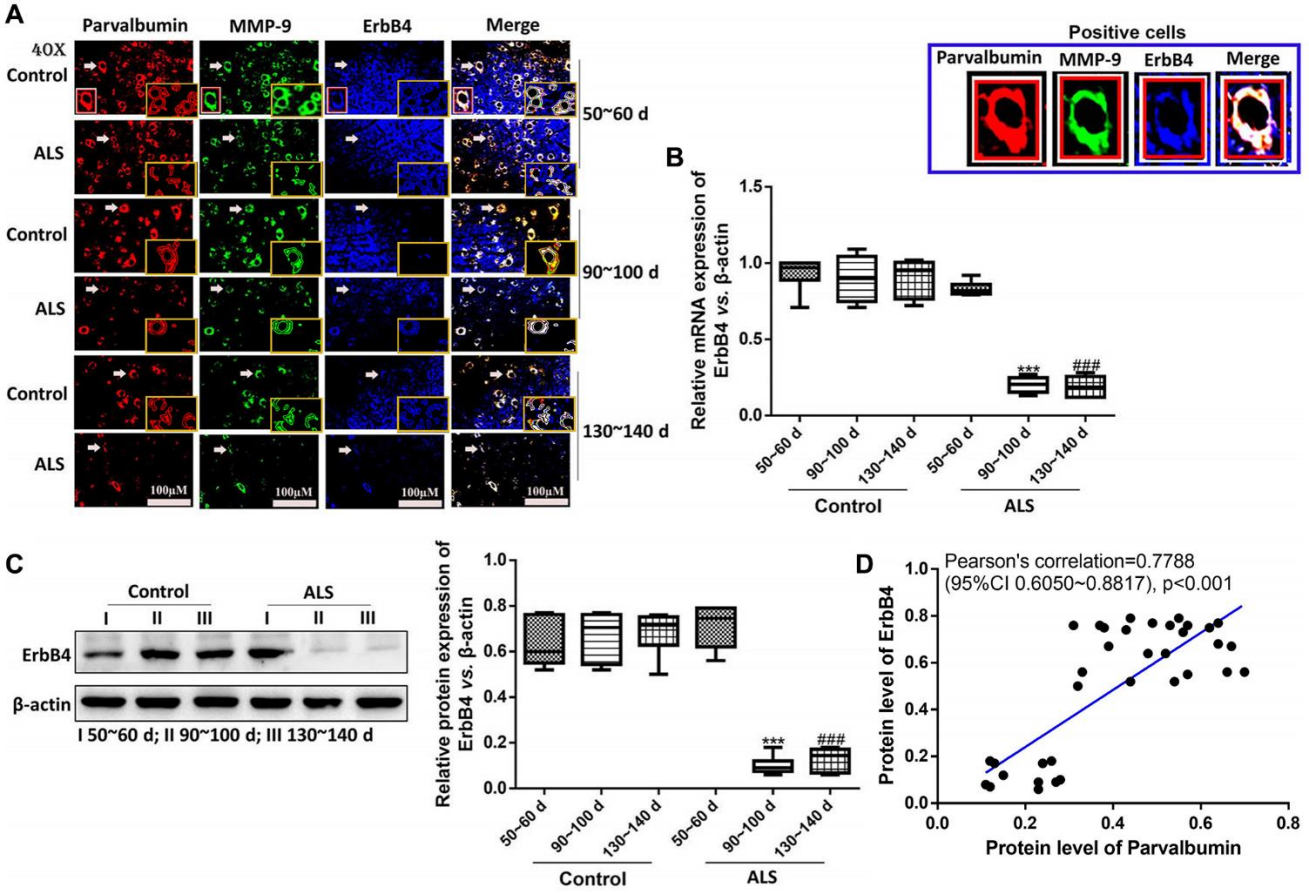
**The expression of ErbB4 was positively correlated with PV interneurons in the spinal cord of ALS mice by immunofluorescence.** The yellow boxes represent the typical positive cells (enlarged approximate twice on the original position). The typical PV, MMP-9 and ErBb4 positive cells are enlarged approximately 4 times and shown on the top right panel. Scale bar = 100 μM (**A**). The expression of ErbB4 was tested by RT-qPCR (**B**) and western blotting (**C**). Pearson’s correlation for protein levels of PV and ErbB4 in all animals (control and ALS) (**D**). ^***^P < 0.001 vs. control at 90~100 days. ^###^*P* < 0.001 vs. control at 130~140 days. In all experiments, half male and half female mice were used in each group (*n* = 6).

Next, we analyzed the expression of ErbB4, a protein which regulates the expression of PV [32], in ALS mice using immunofluorescence. It was found that the expression of ErbB4 was also gradually decreased along with the onset time (Figure 5A–5D). Besides, the levels of ErbB4 were markedly decreased in ALS mice at both symptomatic phase (90~100 days) and symptomatic progression (130~140 days) compared with the control mice (both *P* < 0.05). Pearson’s analysis showed the expression of ErbB4 was positively correlated with PV interneurons in the spinal cord of ALS mice.

### Recombinant NRG1 improved the motor function and enhanced the levels of ErbB4, PV and MMP-9 in ALS mice

**Figure 6:**
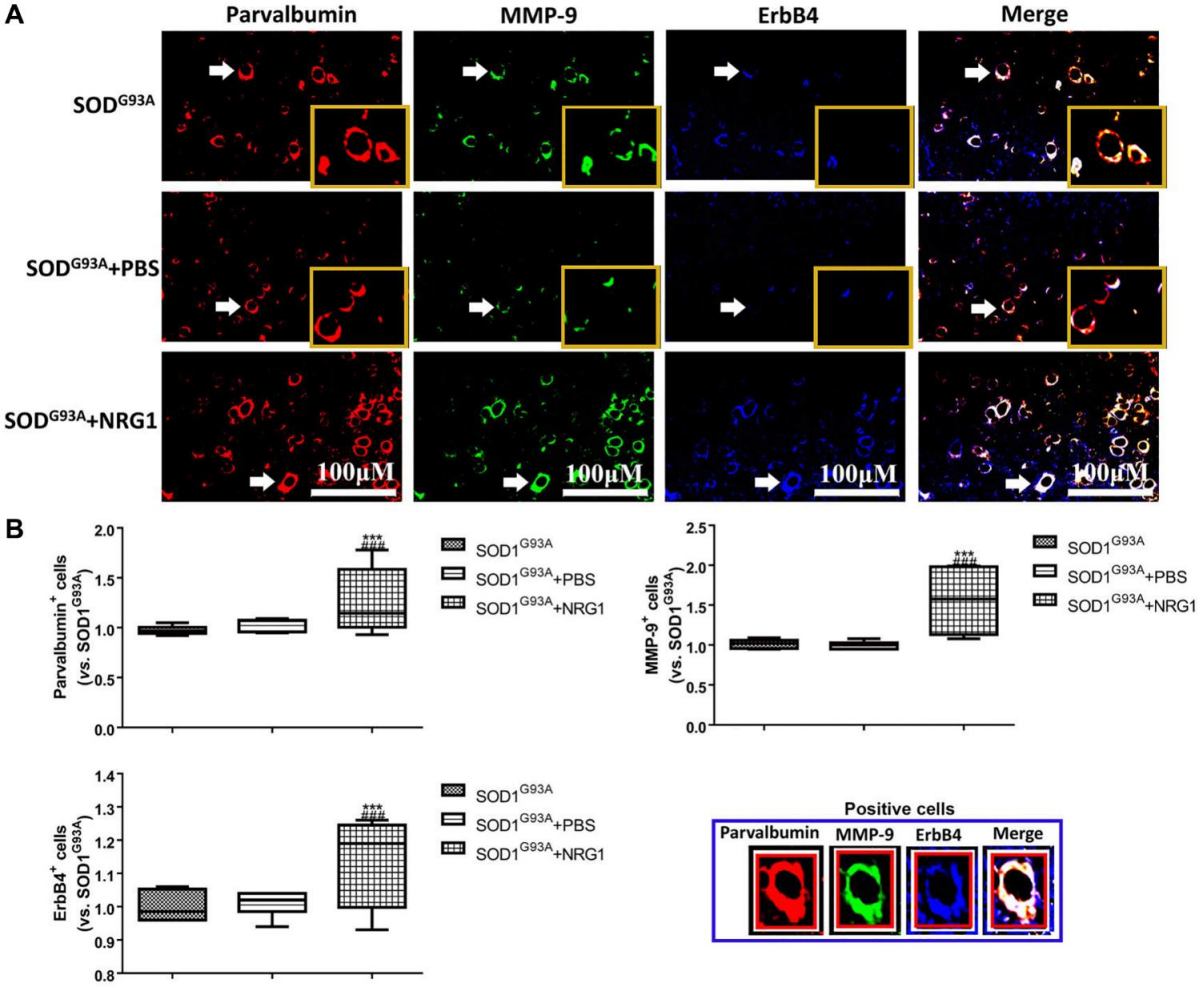
**Recombinant NRG1 enhanced the levels of ErbB4, PV and MMP-9 in ALS mice.** The expression of ErbB4, PV and MMP-9 were determined by immunofluorescence in control mice and mice treated with 0.5 mg/kg NRG1. The yellow boxes represent the typical positive cells (enlarged approximate twice on the original position). The typical PV, MMP-9 and ErBb4 positive cells are enlarged approximate 4 times and shown on the bottom right panel. Scale bar = 100 μM (**A**) and the quantified results (**B**) in mice at 130~140 days. ^***^P < 0.001 vs. SOD1^G93A^. ^###^*P* < 0.001 vs. SOD1^G93A^+PBS. In all experiments, half male and half female mice were used in each group (*n* = 6).

Then the mice were treated with different doses of recombinant NRG1 and rotarod test was conducted to show the change of motor function. We observed that NRG1 could enhance the expression of PV, ErbB4 and MMP-9 in a dose-dependent manner in ALS mice at 130~140 days (Supplementary Figure 3). Next, we also detected the expression of ErbB4, PV and MMP-9 using immunofluorescence in control mice and mice treated with the highest dose of NRG1. The results showed that the expression levels of ErbB4, PV and MMP-9 were all remarkably increased by treatment of recombinant NRG1 compared with the control mice (all *P* < 0.05, Figure 6A, 6B).

**Figure 7:**
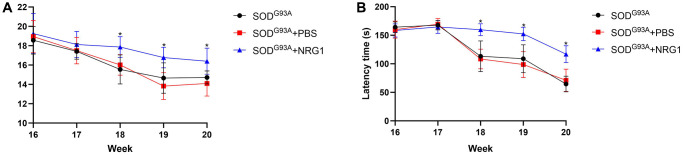
**Recombinant NRG1 enhanced the motor function of ALS mice.** We used the highest dose of NRG1 (0.5 mg/kg) and evaluated the motor function. (**A**) The weight of the ALS mice. (**B**) The latency time of rotarod test. ^*^P < 0.05. In all experiments, half male and half female mice were in each group (*n* = 6).

Then we chose the highest does of NRG1 and evaluated the motor function. As shown in Figure 7A, 7B, we found that from 17 weeks after born, the weight of the ALS mice started to decrease. The mean weight of mice in ALS group treated with NRG1 was markedly higher compared with the control mice from 18 weeks to 20 weeks (all *P* < 0.05). Similarly, the latency time of rotarod test in mice treated with NRG1 was significantly longer than the model mice (all *P* < 0.05).

## DISCUSSION

Despite years’ development, the understanding for ALS is still superficial. In recent years, some studies notice the role of PV interneurons in ALS. However, the detailed changes of PV interneurons ALS spinal cord and the related mechanisms are still unclear. In the present study, we demonstrated for the first time that PV interneurons were gradually decreased in the spinal cord of ALS mice along with the onset time, which was also along with the decrease of FαMNs and ErbB4 levels.

Several studies already reported the potential role of PV interneurons in ALS. In early studies, the loss of PV might promote cytosolic calcium accumulation in motoneurons in ALS, while overexpression PV might delay ALS onset [39, 40]. In recent years, Quarta et al. demonstrated that the restoration of PV-positive neurons in hippocampal CA1 could rescue the long-term potentiation in ALS mice [41]. In another work, it was demonstrated that the activity of PV interneurons was reduced in the late pre-symptomatic ALS [42]. In cerebrospinal fluid of multiple sclerosis patients, it was also found that the expression of parvalbumin was obviously decreased, along with severity of inflammation [43]. A more recent study found that PV interneurons were reduced in post-mortem amyotrophic lateral sclerosis frontal cortex [44]. However, the detailed changes of PV interneurons in ALS and how it influences ALS development are not clear. In this research, it was found that PV interneurons were gradually decreased along with the disease progression of ALS mice. Besides, we also for the first time found the positively correlation among PV, MMP-9 and ErbB4.

FαMNs play an important role in many neurological diseases, including ALS. Early studies found that FαMNs were more sensitive to degrade ALS, while slow α motor neurons were partly resistant to this injury [45, 46]. These results were in consistent with ours. Some studies demonstrated that some proteins like Kir4.1 could selectively induce the dysfunction of FαMNs [47]. However, no study demonstrated the relationship between FαMNs and PV interneurons. In our research, we observed that the levels of FαMNs were also declined during ALS period and were positively correlated with PV interneurons levels, which might be also a selective regulation. We speculated that the decrease of FαMNs in ALS might be partly due to the impaired calcium buffer by PV interneurons dysfunction [48, 49], which might explain the positive correlation between FαMNs and PV interneurons. However, deeper insights are still needed to demonstrate the underlying relationship between them.

Studies already found ErbB4, which can regulate PV interneurons, plays a key role in ALS. However, its deeper insights in ALS are not fully understood. Generally, ErbB4 is essential to maintain the normal function of PV interneurons, and the defection of ErbB4 may lead to reduce of the levels of activity dependent PV interneurons [32]. In ALS, it was found that the mutation of ErbB4 was associated with ALS incidence [37]. Besides, the decrease of circulating ErbB4 is considered as one signal of impaired signaling function in ALS mice [50]. In a study including 6,500 whole genome sequences in ALS, it was found that >70% of people with respiratory onset ALS harbored ErbB4 insertion [51]. A more recent study observed that ErbB4 mutation might lead to reduced auto-phosphorylation of ErbB4 upon neuregulin-1 (NRG1) stimulation in ALS [36]. However, up to now, no study focused on the relationship between ErbB4 and PV interneurons dysfunction in ALS. It this study, we found that ErbB4 was also decreased along with the ALS onset, which was positively correlated with the expression of PV, indicating a potential role of ErbB4 in PV interneurons dysfunction.

Under normal condition, NRG1-ErbB4 signaling is essential for neurotransmitter production and receptor activity and plays an important role for the balance of synaptic microcircuits between PV interneurons and pyramidal neurons [52]. Besides, NRG1-ErbB4 signaling also protects neuron from glutamate induced cell death, which can be reversed by GABA receptor antagonist [53]. ErbB4 is also highly expressed in mice spinal motoneurons and neuromuscular junction, which is essential for the development and regulation of neuromuscular junctions [54]. In ALS, studies demonstrated that NRG1-ErbB4 signaling was associated with both family ALS and sporadic ALS, as well as spinal motoneuron in SOD1 mutation mice [55, 56]. It was found that the inhibition of NRG1-ErbB4 signaling was a key factor which might influence the incidence of ALS, and activation ErbB4 using NRG1 or its agonist might be a potential therapeutic strategy for ALS treatment [37]. In our study, we also found that treatment of NRG1 significantly enhanced the expression of ErbB4, PV and MMP-9, indicating the improvement of MNs in ALS mice.

## CONCLUSION

In summary, PV interneurons are gradually decreased in ALS mice along with the onset time, along with the decrease of FαMNs and ErbB4 levels, indicating that the involvement of PV interneurons in ALS is associated with FαMNs decrease and ErbB4 inhibition. This study might provide a novel direction for the development of ALS.

The study also has some limitations. First, we didn’t measure the mRNA expression of ErbB4 in the tissue samples. The underlying relationship between ErbB4 and PV interneurons were also not clear. Besides, the signaling pathways which may regulate the progression of PV interneurons during ALS need more studies to illustrate.

## Supplementary Materials

Supplementary Figures
