# Supplementary material for: Parvalbumin interneurons dysfunction is potentially associated with FαMNs decrease and NRG1-ErbB4 signaling inhibition in spinal cord in amyotrophic lateral sclerosis
Source: Aging (Albany NY). 2023 Dec 28;15(24):15324–39. doi: 10.18632/aging.205351 (PMC10781496; doi:10.18632/aging.205351)
Supplement: Supplementary Figures [file aging-15-205351-s001.pdf]

SUPPLEMENTARY FIGURES

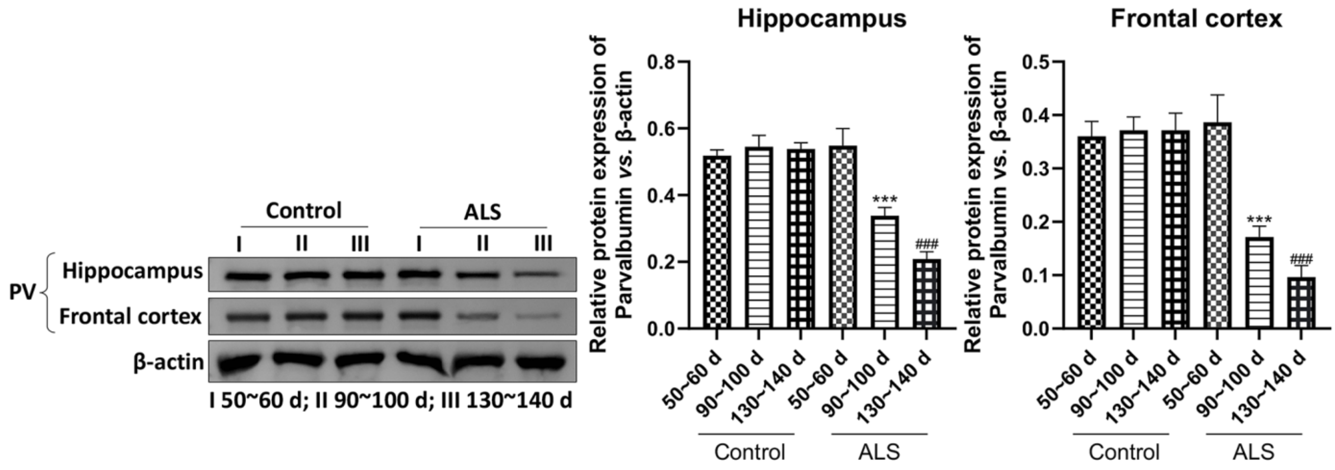

Supplementary Figure 1. Western blotting assay for PV in frontal cortex and hippocampal tissue in control mice of ALS mice at different periods. $^{***}P < 0.001$ vs. control at 90~100 days. $^{\#\#\#}P < 0.001$ vs. control at 130~140 days. In all experiments, half male and half female mice were in each group (*n* = 6).

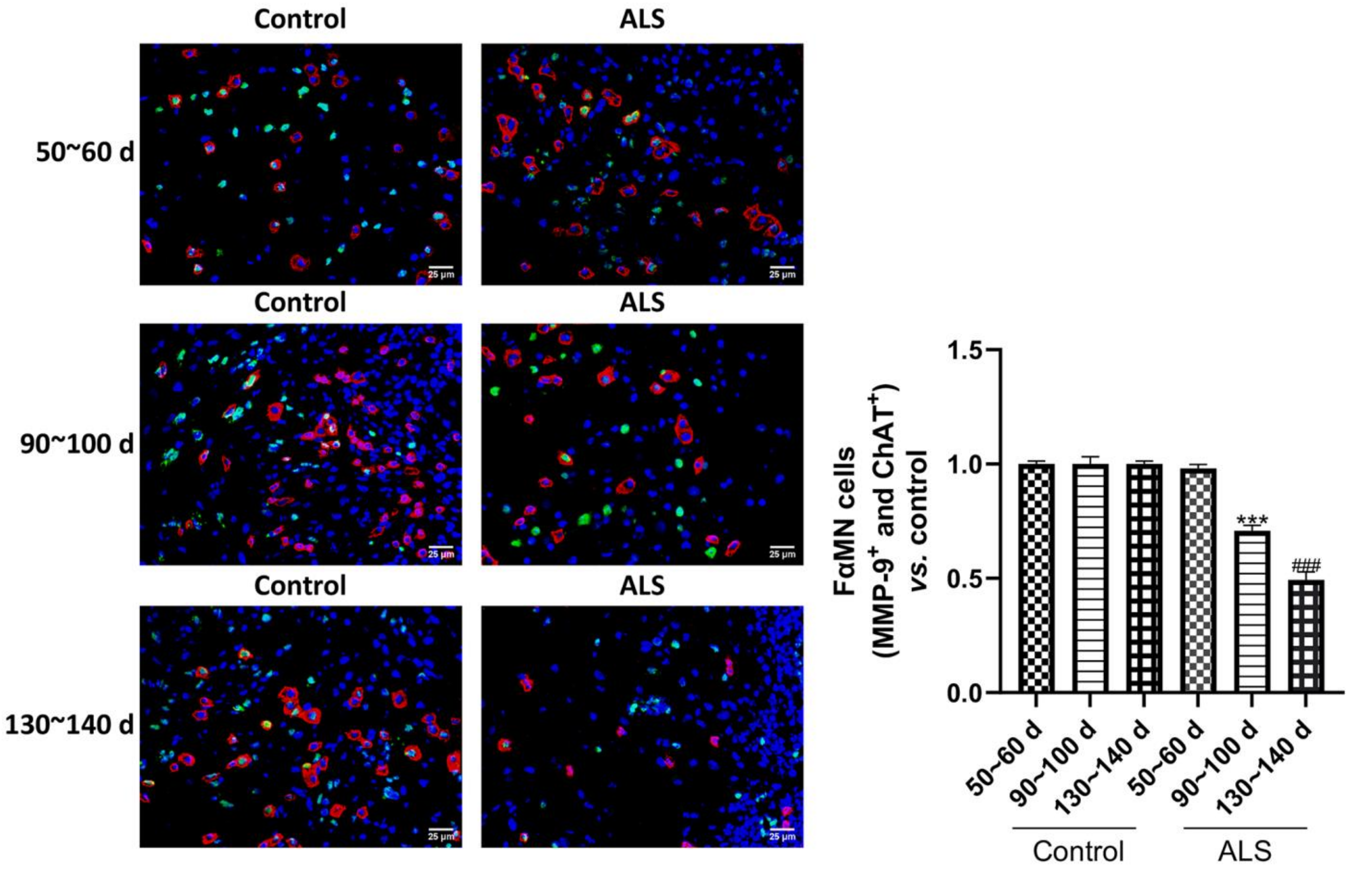

Supplementary Figure 2. Immunofluorescence of FαMNs with marker of MMP-9+ and CHAT-GFP+ in at different periods. $^{***}P < 0.001$ vs. control at 90~100 days. $^{\#\#\#}P < 0.001$ vs. control at 130~140 days. In all experiments, half male and half female mice were in each group (*n* = 6).

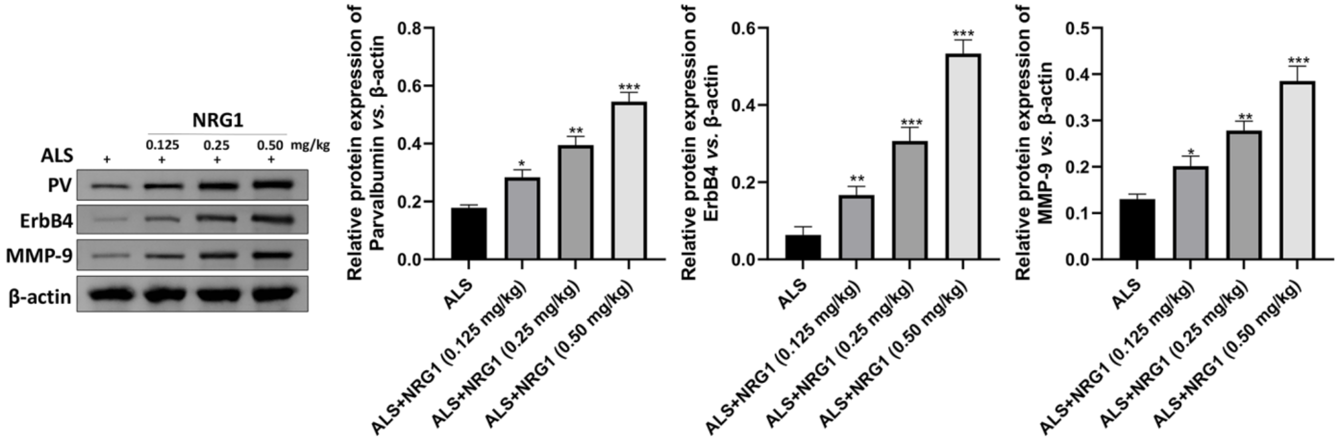

Supplementary Figure 3. Western blotting assay for expression of PV, ErbB4 and MMP-9 in ALS mice treated with different dose of NGR1 at 130~140 days. $^{***}P < 0.001$ vs. control. In all experiments, half male and half female mice were in each group (n = 6).
